# Advances in the Application of Surface-Enhanced Raman Spectroscopy for Quality Control of Cereal Foods

**DOI:** 10.3390/foods14203551

**Published:** 2025-10-18

**Authors:** Pan Meng, Min Sha, Zhengyong Zhang

**Affiliations:** 1 School of Management Science and Engineering, Nanjing University of Finance and Economics, Nanjing 210023, China; 1120241206@stu.nufe.edu.cn (P.M.); minsha@nufe.edu.cn (M.S.); 2 Humanities and Social Sciences Laboratory of Jiangsu Province—Food Safety and National Strategic Governance, Jiangnan University, Wuxi 214122, China; 3 Key Laboratory of Food Processing and Quality Control, Nanjing University of Finance and Economics, Nanjing 210023, China

**Keywords:** surface-enhanced Raman spectroscopy (SERS), cereal foods, quality control, substrate, microbial contamination, pesticide residue

## Abstract

Cereal foods occupy a central position in the global food consumption structure. Staple foods such as wheat, rice, and corn provide essential nutrients like carbohydrates and proteins for billions of people. Long-term intake of these foods can reduce the risk of cardiovascular and cerebrovascular diseases. However, the development of modern agriculture has caused some quality and safety issues such as pesticide residues, mycotoxin contamination, heavy metal residues, and illegal additives in the production, processing, and storage of cereal foods. Traditional detection techniques such as chromatography and mass spectroscopy have limitations including time-consuming procedures and high costs. Surface-enhanced Raman spectroscopy (SERS), with advantages of non-complex pretreatment, rapid detection, and high sensitivity, can accurately detect factors affecting cereal quality. This paper reviews the principles and substrate types of SERS first. Secondly, it systematically summarizes the research progress in the applications of SERS technology in cereal quality control from multiple aspects, including the detection of microbial contamination, pesticide residues, and heavy metal residues. Finally, it provides an outlook on SERS technology. SERS is expected to further improve the accuracy and efficiency of quality control for cereal foods through the development of new substrates, combination with other detection technologies, and intelligent data analysis methods.

## 1. Introduction

Cereal foods refer to foods mainly composed of seeds of gramineous plants, which occupy a pivotal position in the global food consumption structure [1]. Global cereal production is expected to reach 2.91 billion tons in 2025, which is 2.1% higher than that in 2024. Among them, wheat, rice, and corn are three staple foods for billions of people, and the production of these three crops accounts for approximately 90% of total cereal production. In 2024, the global production of wheat, rice, and corn were 0.79 billion tons, 0.52 billion tons, and 1.23 billion tons, respectively [2]. In terms of other common cereals, according to data from the official website of the United States Department of Agriculture (USDA), the global barley production for the 2024/25 season is projected to drop to 143.33 million tons, marking the lowest level in nearly a decade. Sorghum production is expected to reach 63.42 million tons, representing a 7% increase from the previous year. Oat production is anticipated to hit 22.59 million tons, up 16% year-on-year. Millet production is forecast to be 29.17 million tons, a 3% decrease compared to the previous year. Rye production is estimated to stand at 10.6 million tons, down 10% from the previous year. Cereals are rich in carbohydrates, proteins, vitamins, dietary fiber, and minerals, which support the daily physiological activities and health of the human body [3]. Long-term intake of cereal foods can also reduce the risk of cardiovascular diseases, cerebrovascular diseases, cancers, obesity, and diabetes [4,5]. Meanwhile, cereals are also basic raw materials for many processed foods in the food industry, widely used in the production of bread, biscuits, rice noodles, wines, and other foods [6,7,8].

However, with the large-scale development of modern agriculture and the complexity of the food industry chain, cereals face many quality and safety hazards in the long supply chain from the field to the table. For example, they may be contaminated by pesticide and chemical fertilizer residues during planting [9,10], excessive processing may lead to nutrient loss and harmful substances during processing [11,12], and they are susceptible to mycotoxins and pest infestations during shipment and storage [13,14]. These quality problems will not only affect the edible quality and nutritional value of cereals but also may pose serious threats to consumers’ health. For instance, long-term intake of mycotoxins will increase the risk of cancer [15], heavy metal accumulation will cause chronic poisoning [16], and pesticide residues may lead to nervous system damage [17]. Therefore, the strict and efficient control of cereal food quality is of great importance. The Codex Alimentarius Commission (CAC), jointly established by the Food and Agriculture Organization (FAO) and the World Health Organization (WHO), has formulated a series of globally influential standards and guidelines [18], playing a paramount role in harmonizing national cereal quality regulations and protecting public health [19]. The CAC has promulgated specific standards for various cereal products, such as the Standard for Rice (CXS 198-2019) [20], Standard for Maize (CXS 153-2019) [21], Standard for Wheat Flour (CXS 152-2023) [22], and Standard for Oats (CXS 201-2019) [23]. Furthermore, these standards underwent revisions in 2019 and 2023, respectively, to align with contemporary quality paradigms.

Under such circumstances, the development of rapid and reliable technologies for detecting food safety and quality is extremely urgent. Commonly used determination methods mainly include chromatography, mass spectrometry, and their combination [24]. However, these methods require expensive instruments, well-trained operators, and are relatively time-consuming, making them unsuitable for on-site rapid detection of samples [25].

**Figure 1:**
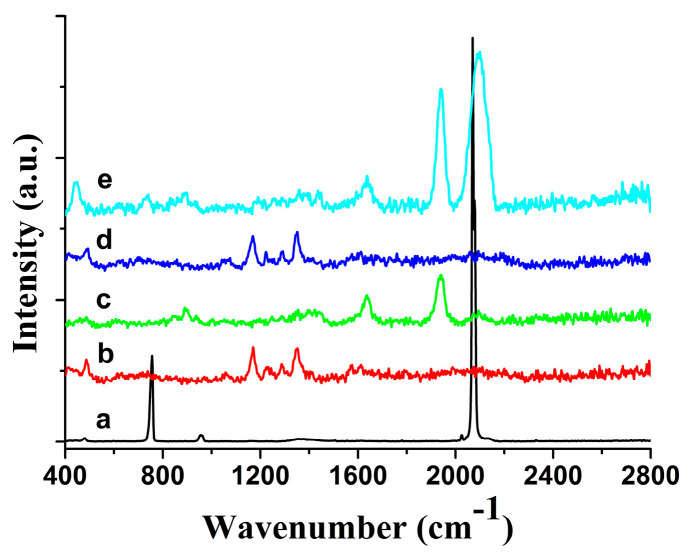
Raman spectra of sodium thiocyanate on an agarose microchip. (**a**) Normal Raman spectrum of the sodium thiocyanate powder; (**b**) normal Raman background spectrum of the agarose microchip; (**c**) normal Raman spectrum of the agarose microchip-based substrate; (**d**) 5 × 10^−6^ g per milliliter sodium thiocyanate on the agarose microchip; (**e**) surface-enhanced Raman spectroscopy spectrum of 5 × 10^−6^ g per milliliter sodium thiocyanate on the microchip-based substrate [26].

As an advanced spectroscopic analysis technology, surface-enhanced Raman spectroscopy (SERS) has shown unique advantages in the field of food safety detection in recent years (Figure 1). Based on the Raman scattering effect, SERS can achieve highly sensitive detection of trace substances by enhancing the Raman signal of target molecules on rough metal surfaces or nanostructures [26,27]. Compared with traditional detection methods, SERS has the advantages of simple operation, fast analysis speed, no need for complex sample pretreatment, and in situ non-destructive detection [28,29].

In the field of cereal food quality control, the importance of SERS technology is increasingly prominent. It can quickly and accurately detect various food contaminants such as pesticides [30], bacteria [31], mycotoxins [32], allergens [33], and even microplastics [34], as well as identify and evaluate the category, origin, and quality of cereals [35]. With its high sensitivity and specificity, SERS can reveal the chemical composition and structural information of analytes at the molecular level [36], providing an efficient and accurate detection method for cereal food quality control. Consequently, it helps to control the quality of cereal foods, discover quality and safety hazards in a timely manner, and promote the high-quality development of the cereal food industry.

## 2. SERS Technology

### 2.1. Enhancement Mechanism of SERS

When light irradiates a substance, it exchanges energy with the substance, producing inelastic scattered light, which is Raman scattering [37]. The peak frequency of Raman scattering corresponds to the vibration mode of functional groups in molecules, and functional groups in different molecules are different. Therefore, each molecule has a unique and definite Raman spectrum [38]. Raman spectroscopy can be used to detect the structural information of substances, thereby achieving the purpose of rapid detection of target substances [39]. However, due to the weak Raman scattering signal, its practical application is greatly limited [40].

In 1974, Fleischman et al. found that the adsorption of pyridine on rough silver electrode surfaces can enhance their Raman scattering signals [41]. Later, many scientists found that the phenomenon of enhanced Raman signals could also be observed on the rough surfaces of gold, copper, and other metals. Through systematic verification, scientists called these phenomena surface-enhanced Raman scattering, and the corresponding spectrum was called the surface-enhanced Raman spectrum [42].

At present, there is no unified view on the enhancement mechanism of SERS. The electromagnetic enhancement mechanism (EM) and chemical enhancement mechanism (CM) are two widely recognized mechanisms for SERS signal enhancement [43]. The EM mainly explains the SERS signal-enhancement mechanism from the perspective of the light, electricity, and magnetic field [44]. When incident light irradiates the surface of an SERS-active substrate, it excites surface plasmon resonance, thereby generating an easily dissipated electromagnetic field on the substrate surface, leading to the significantly amplified and enhanced scattering signal of molecules adsorbed on the substrate surface. The intensity of the enhanced Raman signal is proportional to the square of the amplitude of the excited electromagnetic field [45]. Different from the EM, the generation mechanism of the CM is that a chemical reaction occurs between the analyte molecules in the ground state and the SERS substrate. A resonance excitation of charge transfer states is generated between the reacted molecules and the SERS substrate. The resonance enhancement is derived from the high electronic state excitation of the reacted molecules [46]. Since the SERS phenomenon is related to factors such as the wavelength of incident light, the properties of the substrate, and the adsorption state of the target molecule [47], most SERS phenomena cannot be explained by the EM or CM alone, and both mechanisms need to be used synergistically [48].

### 2.2. SERS Substrates

The enhancement of the SERS signal is directly related to the design and preparation of its substrate [49]. Therefore, the core of SERS technology lies in the preparation of the substrate. An ideal SERS substrate should have both an excellent enhancement effect and good uniformity to ensure that it cannot only capture the weak signal of trace analytes but also ensure the reproducibility of the signal [50]. Commonly used materials for SERS substrates include metal nanomaterials such as gold, silver, copper, etc., and non-metallic nanomaterials such as semiconductors and graphene, etc. Among them, gold and silver nanomaterials are the most common substrate materials due to their chemical stability, low optical loss, and resonance frequency in the visible and near-infrared regions [40]. To achieve ideal performance, various strategies are used to prepare SERS substrates, and the SERS performance of substrates can be regulated by adjusting specific parameters of their structures to meet different detection needs. At present, mainstream SERS substrates can be divided into three categories: colloidal substrates, solid substrates, and flexible substrates.

The use of colloidal substrates is the most direct way to obtain Raman-enhanced signals. Metals such as gold, silver, platinum, copper, and aluminum can be prepared into such substrates, which have good stability and are easy to prepare [51]. Chemical reduction is a common method for preparing colloidal substrates, mainly by reducing metal ions in metal salt solutions to synthesize metal nanoparticles. Relevant studies found that the Raman enhancement effect of colloidal substrates is affected by the type of metal, the size, and surface morphology of nanoparticles [52]. Therefore, appropriate nanoparticles can be synthesized by adjusting these parameters to optimize SERS performance. Muntean et al. synthesized gold and silver nanoparticle colloids by the chemical reduction method, which were self-assembled into films by convection as SERS substrates for detecting propranolol residues in water. A detection limit of 10^−7^ mol/L was achieved on the gold nanoparticle film, and the Raman-enhancement signal of the gold substrate was 10 times higher than that of the silver substrate, and the adsorption performance was improved by 23%. This verified that different metal types, nanoparticle sizes, and surface morphologies would significantly affect the Raman enhancement effect. The results of electromagnetic simulation and density functional theory calculations showed that SERS performance could be optimized by adjusting these parameters, providing an experimental basis for the design of colloidal substrates [53].

Solid substrates mainly use self-assembly technology or lithography technology to fix metal nanoparticles on them, realizing more precise control of the adsorption positions of target analytes, thereby obtaining better uniformity and stability. Self-assembling chemically synthesized nanoparticles into solid substrates is one of the most commonly used methods at present. Self-assembly technology uses surface functional groups and morphological control of nanostructures to form ordered structures, and can prepare high-density, ordered, and controllable nano-arrays [54]. Zhang prepared an ordered single-layer film of silver nanoparticles by self-assembly technology, and the obtained SERS substrate had a good Raman-enhancement ability and reproducibility. The detection limit for methyl parathion could reach 10^−7^ mol/L [55].

**Figure 2:**
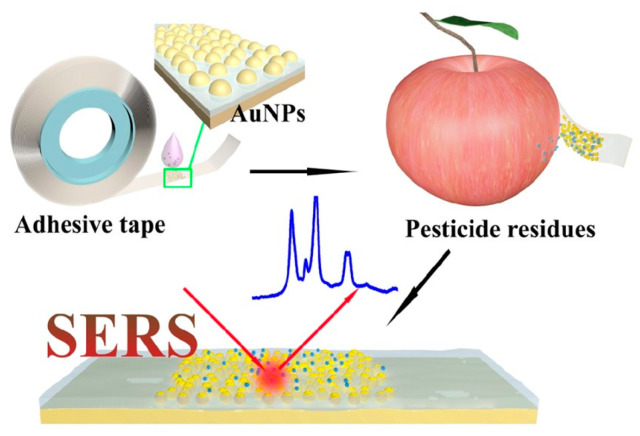
Schematic illustration of the fabrication of SERS tape and extraction of targets from fruit peel surface (apple) for SERS analysis. Adapted with permission from the American Chemical Society and the original author [58].

For SERS detection of trace chemicals on food surfaces, flexible materials such as paper, tape, and polymer materials can also be used to make SERS substrates, which have special application characteristics such as high flexibility, small size, low cost, and convenience for on-site detection [56]. Compared with rigid substrates, which present limitations for the direct on-site detection of analyte residues on irregular sample surfaces, flexible substrates can be directly applied by wiping them across or attaching them to the sample surface [57]. In addition, flexible substrates can be made into any form or size and can be easily wiped cross or wrapped around the sample surface, thus realizing the non-destructive detection of samples. Chen et al. fixed colloidal gold nanoparticles on the tape surface to prepare a flexible substrate for the detection of pesticide residues such as parathion-methyl, thiram, and chlorpyrifos in real samples with complex surfaces including green vegetables, cucumbers, oranges, and apples. The utility of SERS tape was demonstrated by directly extracting pesticide residues in fruits and vegetables via a simple and viable “paste and peel off” approach (Figure 2). Specifically, the detection limits of this method were 2.60 ng/cm^2^ for parathion-methyl, 0.24 ng/cm^2^ for thiram, and 3.51 ng/cm^2^ for chlorpyrifos. Compared with previous work, SERS tape shows impressive sampling efficiency on peels [58].

## 3. Research Progress in the Application of SERS Technology in the Quality Control of Cereal Foods

### 3.1. Detection of Microbial Contamination

Microbial contamination is one of the primary hazards threatening the safety of cereal foods, mainly mold and bacteria. Among them, aflatoxins and vomitoxins produced by Aspergillus flavus and Fusarium have strong carcinogenicity and teratogenicity, which are difficult to destroy even after high-temperature processing [59]. Long-term intake of them will cause serious health problems such as liver damage, precocious puberty, and cancer [60,61,62].

In response to these risks, the Codex Committee on Contaminants in Food (CCCF) promulgated the General Standard for Contaminants and Toxins in Food and Feed (CXS 193-1995) in the Codex Alimentarius in 1995, which underwent its latest revision in 2024. This standard contains the main principles which are recommended by the Codex Alimentarius in dealing with contaminants and toxins in food and feed and lists the maximum levels of contaminants and natural toxicants in food and feed which are recommended by the CAC to be applied to commodities moving in international trade [63].

Specifically, regarding microbial contaminant residues, this standard defines the permissible maximum levels in various cereal foods. For instance, the maximum level for Aflatoxin (Aflatoxin B1 + B2 + G1 + G2) residues are set at 10 μg/kg in maize flour, 5 μg/kg in polished rice, 10 μg/kg in sorghum grain, and 5 μg/kg in cereal-based foods for infants and young children. For Deoxynivalenol residues, the maximum level is set at 2000 μg/kg in cereal grains (wheat, maize, and barley) and 200 μg/kg in cereal-based foods for infants and young children. For Fumonisin (Fumonisin B1 + B2) residues, the maximum level is defined as 4000 μg/kg in raw maize grain and 2000 μg/kg in maize flour and maize meal. In addition to the CAC, many nations have also established regulatory limits for microbial contaminant residues in cereal foods. For example, China has set maximum residue limit (MRL) for mycotoxins in the Chinese national standard GB 2761–2017, including 5 μg/kg for Aflatoxin B1 in wheat and barley, 60 μg/kg for Zearalenone in maize and wheat, and 1000 μg/kg for Deoxynivalenol in maize, wheat, and barley [64]. Likewise, the European Commission permits a maximum allowable content of 2 μg/kg for Aflatoxin B1 in all grains and cereals [65]. By setting strict maximum levels for microbial contaminants, these standards provide essential guidance for the detecting methods.

Aflatoxin B1 (AFB1) is a highly toxic secondary metabolite produced by Aspergillus, which is highly carcinogenic even at trace levels [66], and is widely acknowledged as the most prevalent and toxic fungal toxin among the various mycotoxins [67]. In recent years, SERS technology has been widely used in AFB1 detection due to its high sensitivity [68]. In the study by Jiao et al. [69], 5-aminotetramethylrhodamine-modified gold–silver core–shell nanoparticles were used as SERS substrates, and the SERS signal of AFB1 was selectively quenched by the Hg^2+^-mediated aggregation effect. By optimizing the sample pretreatment method and collecting the characteristic peak at 1500 cm^−1^, a standard curve of AFB1 was established for quantitative analysis, achieving an ultra-low detection limit of 0.03 ng/mL in wheat samples. Han et al. developed an aptamer-based detection method, using an Fe_3_O_4_@Au nanocomposite with a satellite structure as the substrate; AFB1 aptamers and their complementary DNA were modified on magnetic nanoparticles and silver nanoparticles, respectively. Aggregation was induced through DNA hybridization to form “hotspots”, and the rapid detection of AFB1 in coix seeds was realized when combined with magnetic separation technology [70]. This technology accurately quantified AFB1 by monitoring the characteristic peak of the aptamer at 735 cm^−1^ and combining multivariate statistical analysis methods, with a detection limit of 0.006 ng/mL.

Deoxynivalenol (DON) is a type B trichothecene mycotoxin produced by the genus Fusarium, with strong immunotoxicity and intestinal toxicity [71]. Recently, significant progress has been made in the detection of DON based on SERS [72]. Zhao et al. developed a bionic magnetic SERS aptasensor based on “dual-antenna” nano-silver, using β-cyclodextrin-coated silver nanoparticles (β-CD@AgNPs) as the SERS substrate [73]. β-CD@AgNPs was modified by 4-mercaptobenzoic acid (4-MBA) and the aptamers, respectively, with chemical bonds and host–guest interactions, which was reflected in the “dual antennae” characteristics of identifying target and SERS signal labels. In addition, Fe_3_O_4_@Au-cDNA was used as a capture probe, enabling fast magnetic separation and enhanced Raman scattering. In the presence of DON, its competitive binding with the aptamer led to the dissociation of the probe complex, and the SERS signal weakened after magnetic separation, realizing ultra-sensitive detection of DON in corn flour with a detection limit of 0.032 pg/mL. In contrast, Wang et al. used colloidal gold nanoparticles as SERS substrates to directly capture the cellular components of fungi such as Aspergillus niger and Fusarium to obtain characteristic peaks in their fingerprint spectra at 623 cm^−1^ and 728 cm^−1^. By combining principal component analysis and linear discriminant analysis, the rapid identification of toxin-producing fungi in corn was achieved [74]. Although this method did not directly detect DON, it indirectly predicted the risk of DON contamination by monitoring toxin-producing fungi. The two studies used SERS technology from two dimensions of directly detecting the DON toxin and indirectly monitoring toxin-producing fungi, providing highly sensitive technical solutions for DON contamination control in cereals.

Zearalenone (ZEN) is an estrogen-like mycotoxin produced by Fusarium, whose target organ is mainly the reproductive system of female animals, and also has certain effects on male animals. In case of acute poisoning, it will have certain toxic effects on the nervous system, heart, kidneys, liver, and lungs [75]. Li et al. developed a cauliflower-like three-dimensional SERS substrate, constructing a porous structure by self-assembling gold nanoparticles, realizing the label-free simultaneous detection of ZEN and other mycotoxins in corn [76]. In this study, corn samples were extracted with a mixed solvent of acetonitrile and water, centrifuged and filtered, and then directly dropped onto the substrate. SERS spectra in the wavelength range from 500 to 1800 cm^−1^ were collected using a 785 nm excitation wavelength laser. Qualitative analysis was performed through the characteristic peaks of ZEN at 1002 cm^−1^ and 1605 cm^−1^, and quantitative analysis was conducted by combining with a partial least squares regression model, with a detection limit of 24.8 ng/mL. Zhang et al. developed a multiplex SERS-based lateral flow immunosensor with dual Raman labels, using gold–silver core–shell nanoparticles (Au@Ag NPs) and 4-MBA/5,5′-dithiobis (2-nitrobenzoic acid) (DTNB) as signal tags, realizing the detection of ZEN in corn samples through competitive immune reactions [77]. After the sample was extracted with 70% methanol, it was mixed with antibody-modified nano-probes for chromatography. SERS signals were collected at the test line of the test strip, and environmental interference was eliminated through calculating the ratio of the characteristic peak intensities of DTNB at 1332 cm^−1^ and 4-MBA at 1077 cm^−1^. Quantitative analysis was completed by establishing a standard curve with a detection limit of 0.21 pg/mL.

In terms of the detection of other biotoxins, He et al. developed a dual-mode detection system based on aptamer and gold nanorods for the determination of Fumonisin B1 (FB1) in corn. This system combined SERS and fluorescence spectroscopy for quantitative analysis. The principle was to first fix the complementary DNA (cDNA) of FB1 aptamer on the surface of a platinum-coated gold nanorod substrate and then let the FB1 aptamer modified with fluorescent dye hybridize with cDNA. In the absence of FB1, the aptamer and cDNA associated, producing strong SERS signals and weak fluorescence signals. Conversely, in the presence of FB1, the aptamer dissociated from cDNA and bound to FB1, leading to a decrease in the SERS signal and an increase in the fluorescence signal. This method required no complex sample preparation; corn samples could be analyzed after undergoing grinding, spiking, extraction, centrifugation, filtration, and other treatments. During detection, the SERS signals at 1366 cm^−1^ decreased linearly within the concentration range from 10 to 500 pg/mL, while the fluorescence signals at 670 nm increased linearly in the concentration range from 10 to 250 pg/mL. In analytical chemistry, recovery is a key indicator for assessing the accuracy of a detection method. It represents the percentage of a standard substance with a known concentration that can be successfully extracted and measured by the entire analytical method after being added to a real sample [78]. In this study, the average recoveries of the method for spiked corn samples ranged from 92% to 107%, which was in good agreement with the results obtained by the liquid chromatography–mass spectrometry method. This method exhibited excellent selectivity for FB1, with no significant interference from other mycotoxins, thus demonstrating favorable practicality and reliability [79]. Khan et al. developed a graphene-Au nanostar nanocomposite SERS substrate [80], using aptamer-based dual-mode detection with both fluorescence and SERS for the T-2 toxin in wheat. After the sample was extracted with acetonitrile, specific recognition by the aptamer resulted in fluorescence quenching, and principal component analysis was used to distinguish different concentrations. There was distinct linearity between the T-2 toxin concentration and the dual fluorescence and SERS signals with lower limits of detection of 8.5 × 10^−11^ mol/L and 1.2 × 10^−11^ mol/L, respectively. These studies demonstrate the application of SERS for detecting various major microbial contaminants in different cereal foods (Table 1).

### 3.2. Detection of Pesticide Residues

To reduce crop yield losses and improve food quality, the use of pesticides to prevent and control various agricultural pests, pathogens, and weeds has become an indispensable method in modern agricultural systems [81]. But excessive use of pesticides is harmful to the environment and human body [82].

Considering these food safety threats, the Codex Committee on Pesticide Residues (CCPR) established a series of Codex Maximum Residue Limits (MRLs) for pesticides in various food commodities within the Codex Alimentarius. Until 2024, the Codex Alimentarius Commission adopted 6453 MRLs for different combinations of pesticides and commodities [83]. It also includes MRLs for various cereal foods. For example, the MRL for Paraquat is 0.03 mg/kg in maize and sorghum grain, and 0.05 mg/kg in rice and maize flour. The MRL for 2,4-dichlorophenoxyacetic acid is 2 mg/kg in wheat and rye, 0.1 mg/kg in rice, and 0.05 mg/kg in maize. The MRL for Pentachloronitrobenzene is 0.01 mg/kg in barley, maize, and wheat. To enforce these standards and ensure compliance, the development of simple and effective methods for detecting pesticide residues for the quality control of cereals is crucial.

In the field of detection of fungicides, Neng et al. proposed a method combining SERS with molecularly imprinted polymers (MIPs) for the rapid and sensitive detection of the fungicide pentachloronitrobenzene (PCNB) [84]. Oil-Soluble Ag NP-embedded MIPs were synthesized as SERS substrates to solve the problem of poor water solubility of PCNB, and their uniformity and crystal structure were verified by transmission electron microscopy and X-ray diffraction. In terms of sample preparation, MIPs were prepared by free radical polymerization, with PCNB as the template molecule, methyl methacrylate as the functional monomer, and silver nanoparticles embedded as SERS enhancement materials. Data were collected using a 785 nm excitation wavelength spectrometer, and specific binding of PCNB was ensured by optimizing the adsorption time. In data analysis, the vibration peak of the benzene ring at 1600 cm^−1^ was used as the characteristic signal. The quantitative linear range of the method was from 0.005 to 0.15 μg/mL, with a detection limit as low as 5.0 ng/mL. This method showed high selectivity in rice samples, with a recovery rate from 94.4% to 103.3%, consistent with the results of gas chromatography-mass spectrometry, providing a portable and low-cost solution for pesticide residue detection.

In the field of the detection of herbicides, Xu et al. developed a highly sensitive detection method combining SERS with magnetic molecularly imprinted polymers (Mag@MIPs) for the rapid detection of the herbicide 2,4-dichlorophenoxyacetic acid (2,4-D) in wheat and rice [85]. The Mag@MIP/Au NPs substrate was obtained using gold nanoparticles as signal enhancement probes combined with magnetic molecularly imprinted polymers through electrostatic adsorption. In terms of sample preparation, 2,4-D was used as the template molecule, acrylamide as the functional monomer, and Mag@MIP NPs were synthesized by surface imprinting technology, its structure and morphology were verified by Fourier transform infrared spectroscopy and transmission electron microscopy. Data were collected using a 785 nm excitation wavelength spectrometer, and the adsorption time was 120 min to ensure specific binding of 2,4-D. In data analysis, the characteristic peak at 1071 cm^−1^ was chosen to establish a quantitative linear curve, with the linear range from 10^−1^ to 10^5^ ng/mL, detection limit as low as 0.00147 ng/mL, and recovery rate from 72.7% to 90.9%. Chen et al. constructed a high-performance Au@MIL-101/PMMA/DT SERS substrate based on a metal–organic framework (MOF)-modified Au nanoparticle (Au@MIL-101) array for the highly sensitive and stable detection of paraquat in cereals [86]. First, Au@MIL-101 nanoparticles were closely arranged on a polymethyl methacrylate film by self-assembly technology and fixed on tape to form a solid substrate. The interactions between iron ions in MIL-101 and nitrogen atoms in paraquat were used to adsorb paraquat into the electromagnetic enhancement region of the gold nanoparticles. In terms of sample preparation, the high activity and stability of the substrate were ensured by optimizing the concentration of polymethyl methacrylate and the thickness of the MOF shell. Data analysis showed that the method exhibited an excellent linear range from 10^−8^ to 10^−2^ mol/L, with a detection limit as low as 1.83 μg/kg, and a recovery rate from 91.57% to 102.32% in wheat, corn, and rice. In addition, the substrate has high uniformity and long-term stability, and the signal can remain 90% within two months, providing a reliable tool for the rapid detection of pesticide residues in complex food matrices.

In the field of the detection of insecticides, Weng et al. used SERS combined with chemometrics to achieve highly sensitive detection of pirimiphos-methyl in wheat [87]. Thiolated methoxy polyethylene glycol-modified gold nanorods were synthesized, which significantly enhanced the Raman signal. Sample pretreatment adopted a simplified process of acetone oscillation extraction combined with centrifugation and filtration to effectively separate the target substances. In the data analysis stage, the characteristic peaks were assigned by density functional theory. The Savitzky–Golay derivative method and principal component analysis (PCA) were used for noise reduction, and the performances of partial least squares regression (PLSR), support vector machine regression, and random forest models were compared, the PLSR-PCA combined model was optimal. The detection limit of this method for the extraction solution is as low as 0.2 mg/L, and for actual wheat samples it is as low as 0.25 mg/L. The predicted recovery rate was from 94.12% to 106.63%, and the deviation from the results of gas chromatography–mass spectrometry was only from 0.10% to 6.63%. This method has advantages of portability, high precision, and low detection limit, providing a practical solution for monitoring pesticide residues in cereals (Table 2).

### 3.3. Applications in Other Fields

In the field of the detection of chemical contaminants in food, Droghetti et al. developed a sensor system based on functionalized Ag nanoparticles for detecting benzophenones that may migrate from packaging materials into cereal foods [88]. Two forms of Ag nano substrates, colloidal suspension, and an immobilized silver plate were used in the study, which were functionalized with lucigenin (LG). The N^+^-CH_3_ part of LG was used to generate specific interactions with the CO groups in benzophenone (BP) and 4-methylbenzophenone (4MBP). In terms of sample preparation, spiked oat samples were directly extracted without complex purification steps. During data collection, by measuring the signal intensity after the interaction between the analyte and LG, linear calibration curves between concentrations and signal intensities were established to realize quantitative detection of target substances. The lowest amount of analytes revealed by the SERS method, in the analyte stock solutions and in the spiked breakfast cereal, were 50 μmol/L and 70 μmol/L, respectively. The calculated limit of detection value was 16 μmol/L. Data analysis showed that at higher concentrations (>70 μmol/L), the SERS detection results were in good agreement with gas chromatography–mass spectrometry and mass spectrometry, with an error of less than 10%, and no interference peaks from other compounds were observed, indicating that the method has good selectivity and specificity. Zhao et al. developed an SERS method based on Ag nanoparticles for detecting Sudan Black B in dyed black rice. Acetone was used as the solvent to extract Sudan Black B from dyed black rice without complex purification steps, and silver colloid was used as the SERS substrate. When hydrochloric acid was added to the mixture of sample and silver colloid, the SERS signal could be enhanced. After obtaining the characteristic SERS spectrum of Sudan Black B, quantitative detection was realized using the linear relationship between signal intensities and concentrations. The minimum detection concentration of Sudan Black B in the standard solution by this method was 0.05 mg/L, while the minimum detection content in black rice extract was 0.1 mg/kg. In addition, the effectiveness of the designed extraction method was indirectly verified by studying the relationship between the dyeing time of black rice in the Sudan Black B solution and signal intensities [89]. Studies have shown that the SERS method can quickly and sensitively detect Sudan Black B in black rice, providing a practical means for the detection of food additives in cereal foods.

The General Standard for Contaminants and Toxins in Food and Feed (CXS 193-1995) issued by the CCCF in the Codex Alimentarius also stipulates the maximum levels of permissible heavy metal residues in various cereal foods. For example, the maximum level of cadmium residue is 0.1 mg/kg in cereal grains, 0.2 mg/kg in wheat, 0.4 mg/kg in rice, and 0.15 mg/kg in quinoa. To detect heavy metal residues in cereal foods, Zuo et al. developed an SERS-based quantitative detection method for the highly sensitive analysis of cadmium ions (Cd^2+^) in rice [90]. Au nanoparticles modified with trimercaptotriazine (TMT) were used as the substrate, and ratiometric Raman signal changes were generated through the specific binding between TMT and Cd^2+^. In sample preparation, rice was digested by microwave and mixed with TMT-AuNPs probes, and a masking agent was added to eliminate the interference of Pb^2+^. A conical holed substrate was used to further enhance SERS signals, and the signal intensity after multiple reflections was 5 to 10 times higher than that of the plane substrates because of the internal multiple reflections of both the excitation laser beam and the Raman scattering photons within conical holes. In data analysis, a calibration model was established by partial least squares regression, realizing the accurate quantification of Cd^2+^ in the range from 0.5 to 100 μg/L, with a detection limit as low as 8 μg/kg, which is lower than the limit in the Chinese national standard. This method was verified in three actual rice samples, with a recovery rate from 93.8% to 109.4%. The results were consistent with those of inductively coupled plasma mass spectrometry, showing its application potential in the rapid screening of heavy metals in cereals.

SERS technology can also be used for the detection of nutrients in cereal foods. Radu et al. developed an analytical method using SERS technology for the simultaneous detection of vitamin B2 (riboflavin) and B12 (cyanocobalamin) in cereal foods [91]. Ag nanoparticles were used as the SERS substrate; the stability of the substrate was verified by optimizing acidic conditions. Enzymatic hydrolysis and acid hydrolysis extraction schemes were compared to improve the extraction efficiency of target vitamins. Data were collected using a confocal Raman spectrometer with a laser wavelength of 488 nm, achieving a detection limit of 10^−7^ mol/L in a breakfast–corn flake food matrix. By analyzing the characteristic peaks of vitamins, the SERS method successfully distinguished the two vitamins and proved its ability for selective detection in complex matrices. This technology shows the advantages of rapidity and low cost, providing a new method for the quality control of cereal foods (Table 3).

## 4. Prospect

In the field of quality control for cereal foods, SERS technology has shown great application potential due to its advantages of high sensitivity and rapid detection. It can accurately identify trace pesticide residues on and inside cereals, judge whether cereals are contaminated by microorganisms, quickly determine heavy metal residues, and efficiently analyze the composition and content of nutrients such as proteins and vitamins in cereals, thereby establishing a robust foundation for ensuring the quality and safety of cereal foods.

However, compared with the application of SERS technology in other fields, there is relatively less research in the field of cereal food quality control, currently focusing on the detection of rice, wheat, and corn. This phenomenon stems from multiple factors. On the one hand, although SERS technology offers distinct advantages, its application in the field of quality control for cereal foods has remained limited due to the maturity of the industrial chain and the widespread use of traditional detection methods, such as high-performance liquid chromatography, gas chromatography–mass spectrometry, and enzyme-linked immunosorbent assays [92], and this situation is expected to improve with the continued development of SERS technology. On the other hand, SERS technology has high requirements for sample purity, but cereal samples have complex matrix components, conventional extraction methods such as ultrasound and oscillation find it difficult to effectively separate the analytes [93]. To reduce matrix interference, several techniques can be employed during sample preparation such as solid phase extraction and immunoaffinity chromatography [94], but these steps will increase the operation’s complexity and may lead to the loss of the analytes [95], which is contrary to the advantage of rapid detection by the SERS method. In addition, the signal intensity of the SERS method is highly dependent on the morphology of the substrate, such as the size and spacing of nanoparticles, while the preparation of substrates is easily affected by batches and processes, resulting in large fluctuations in detection results [96]. For the strict safety standards of cereal foods, this instability caused by poor reproducibility of substrates often makes it difficult to meet the needs of actual testing. Despite the aforementioned drawbacks of SERS technology, its advantages of fast analysis and on-site detection align well with the requirements of cereal food quality control, thereby exhibiting unique application prospects. The future development of SERS technology in the field of cereal food quality control will mainly focus on developing new substrates with good reproducibility [97,98,99,100,101,102,103], combining with other traditional detection technologies [104,105,106,107], and integrating with intelligent data analysis technologies to expand the application boundary and realize wider application [108,109,110,111,112,113,114,115].

The key reason why SERS technology can achieve ultra-high sensitivity detection lies in the significant enhancement effect of the substrate on Raman signals. Therefore, the performance and stability of the substrate will directly affect the reliability and reproducibility of SERS detection results. Future research will focus on developing manufacturing methods that can precisely control nanostructures, such as advanced nanoimprint lithography [97], block copolymer self-assembled substrates [98], and DNA nanotechnology-guided assembled substrates [99] to ensure the uniformity of substrates and high reproducibility of SERS signals. New composite structures, such as core–shell structures [100], core–satellite structures [101], nano-pillar arrays [102], and metal–semiconductor heterostructures [103], can also be designed to obtain higher and more stable enhancement effects.

In the field of complex matrix sample analysis, traditional spectral detection technology often has significant limitations. Although SERS technology has advantages of high sensitivity and rapid detection, it is susceptible to background interference in complex matrices and lacks molecular spatial distribution information [104]. Innovative combinations of SERS technology with other detection technologies can complement and synergistically enhance the advantages of their characteristics. Oksenberg et al. combined SERS with surface-enhanced infrared spectroscopy, proposing a complementary surface-enhanced vibrational spectroscopy method for detecting the vibrational characteristics of single-layer molecules bound to metal substrates through chemical interaction. The study explained the differences in vibrational information between the two surface-enhanced spectroscopies in detecting coupled molecular vibrations, pointing out that the combination of the two methods can cover more comprehensive molecular vibration modes [105]. Amirsalari et al. combined SERS with hyperspectral imaging technology, developing a multimodal microscope system integrating hyperspectral Raman imaging, dark-field scattering imaging, and angular scattering spectroscopy analysis, realizing comprehensive characterization of plasmonic nanostructures, breaking through the spatial resolution limit of SERS technology, and achieving super-resolution imaging of target molecules [106]. Marina et al. combined SERS with mass spectrometry, realizing the rapid screening and structural confirmation of psychotropic drugs in alcoholic beverages. First, SERS was used for in situ preliminary screening, and then mass spectrometry was used to solve the false positive problem in complex matrices, with strong anti-matrix interference abilities and low detection limits [107].

In face of complex and massive SERS spectral data, traditional data processing methods make it difficult to extract key information. With the development of intelligent data analysis technologies in recent years, such as artificial intelligence (AI) and machine learning algorithms, the efficiency of data processing has greatly improved [108]. On the one hand, AI can predict optical properties such as resonance, extinction, and scattering by training machine learning algorithms (such as Gaussian process regression and artificial neural networks) on substrate structure parameters (such as size, shape, and material composition), thereby designing and optimizing SERS substrates [109,110]. On the other hand, using models such as residual network and random forest to analyze spectral data can identify molecular fingerprints from complex spectra and achieve the qualitative detection of trace substances, with an accuracy higher than traditional methods such as principal component analysis [111,112]. In terms of quantitative analysis, combining SERS technology with machine learning algorithms such as partial least squares regression and support vector machines can accurately calculate the concentration of target analytes in samples [113]. The integration of artificial intelligence into SERS can enhance the speed and accuracy of detection [114]. Particularly, machine learning and deep learning can be trained to develop predictive models that correlate specific spectral features with the presence and concentration of contaminants, making accurate predictions based on large datasets. These models can be continuously refined and improved as more data are collected, leading to more precise and reliable detection methods [115].

## Figures and Tables

**Table 1:** Application of SERS in the detection of microbial contamination in cereal foods.

Cereal Categories	Analytes	SERS Substrates	Limit of Detection	Recoveries	Ref.
Wheat	Aflatoxin B1	NH_2_-Rh-Au@Ag CSNPs	0.03 ng/mL	—	[69]
	T-2 toxin	rGO-AuNS	1.2 × 10^−11^ mol/L	—	[80]
Corn	Deoxynivalenol	β-CD@Ag NPs	0.032 pg/mL	88.7–110.58%	[71]
	Toxin-producing fungi	Au NPs	—	—	[74]
	Zearalenone	Au NPs/PDMS@AAO	24.8 ng/mL	—	[76]
	Zearalenone	4-MBA/DTNB-Au@Ag CSNPs	0.21 pg/mL	78.9–106.2%	[77]
	Fumonisin B1	Au NR@Pt	10 pg/mL	92–107%	[79]
Coix seed	Aflatoxin B1	Fe_3_O_4_@Au nanocomposite	0.006 ng/mL	98.2–101.2%	[70]

—: Not reported.

**Table 2:** Application of SERS in the detection of pesticide residues in cereal foods.

Cereal Categories	Analytes	SERS Substrates	Limit of Detection	Quantitative Linear Range	Recoveries	Ref.
Rice	Pentachloronitrobenzene	Oil-Soluble Ag NPs- Embedded MIPs	5.0 ng/mL	0.005–0.15 μg/mL	94.4–103.3%	[84]
Wheat, Rice	2,4-Dichlorophen- Oxyacetic acid	Mag@MIP/Au NPs	0.00147 ng/mL	10^−1^–10^5^ ng/mL	93.5–102.2%	[85]
Wheat, Corn, Rice	Paraquat	Au@MIL-101/PMMA/DT	1.83 μg/kg	10^−8^–10^−2^ mol/L	91.57–102.32%	[86]
Wheat	Pirimiphos-methyl	mPEG-SH-coated GNRs	0.25 mg/L	0.25–23.93 mg/L	94.12–106.63% (Predicted)	[87]

**Table 3:** Application of SERS in other fields of quality control for cereal foods.

Cereal Categories	Analytes	SERS Substrates	Limit of Detection	Quantitative Linear Range	Recoveries	Ref.
Oat	Benzophenone, 4-Methylbenzophenone	Lucigenin-functionalized Ag NPs	16 μmol/L	—	84%	[88]
Black rice	Sudan Black B	Ag NPs	0.1 mg/kg	—	—	[89]
Rice	Cd^2+^	TMT-AuNPs	8 μg/kg	0.5–100 μg/L	93.8–109.4%	[90]
Corn	Vitamin B2, Vitamin B12	—	10^−7^ mol/L	—	—	[91]

—: Not reported.

## Data Availability

No new data were created or analyzed in this study. Data sharing is not applicable to this article.
